# Perceived Consequences of COVID-19 Restrictive Visitation Policy on Family Members at Rural Hospitals in Vhembe District: A Qualitative Study

**DOI:** 10.3390/nursrep13040117

**Published:** 2023-10-07

**Authors:** Maria Sonto Maputle, Takalani Mbedzi, Mary Maluleke, Mutshinyalo Lizzy Netshikweta, Dorah Ursula Ramathuba, Ndidzulafhi Selina Raliphaswa, Takalani Rhoda Luhalima, Seani Adrinah Mulondo, Thivhulawi Malwela, Azwidihwi Rose Tshililo, Julia Langanani Mafumo, Nwamuhohova Hilda Shilubane, Nthomeni Dorah Ndou, Khathutshelo Grace Netshisaulu

**Affiliations:** Department of Advanced Nursing, University of Venda, Private Bag X5050, Thohoyandou 0950, South Africa; takalani.mbedzi@univen.ac.za (T.M.); mary.maluleke@univen.ac.za (M.M.); dorah.ramathuba@univen.ac.za (D.U.R.); ndidzulafhi.raliphaswa@univen.ac.za (N.S.R.); takalani.luhalima@univen.ac.za (T.R.L.); seani.mulondo@univen.ac.za (S.A.M.); thivhulawi.malwela@univen.ac.za (T.M.); rose.tshililo@univen.ac.za (A.R.T.); julia.mafumo@univen.ac.za (J.L.M.); hilda.shilubane@univen.ac.za (N.H.S.); nthomeni.ndou@univen.ac.za (N.D.N.); khathu.netshisaulu@univen.ac.za (K.G.N.)

**Keywords:** communication, consequences of COVID-19, family-centred care, family members, restrictive visitation policy

## Abstract

From a public health standpoint, a stringent visitation policy was necessary during the COVID-19 pandemic, but it had unforeseen communicative and emotional health consequences for family members. This study explored family members’ experiences regarding implementing a restricted visitation policy when a patient was admitted with COVID-19 at public hospitals in the Vhembe district. Researchers used an exploratory, descriptive, and contextual qualitative technique. Twelve family members made up the population. Unstructured telephone interviews were used to obtain the data, and open coding was used to analyse data. Ethics were consistently followed. Before taking part, participants provided verbal informed consent, acknowledging that they could withdraw from the study if necessary. Three themes emerged: inadequate measures for temporary communication channels and techniques, the mental health effects of COVID-19 admission, and poor/lack of standardised visitation policy during the COVID 19-pandemic. There was a need to balance safety from contracting COVID-19 infection and promoting family-centred care. Virtual visits through telecommunication solutions could reduce fear and anxiety as the family could be updated on the progress of the hospitalised relative. Alternatively, hospital managers must allocate a dedicated person in the unit to update families when they call and enquire about the conditions.

## 1. Introduction

In December 2019, Wuhan, China, reported the first case of the contagious illness COVID-19. The World Health Organization later declared a pandemic on the 11th of March 2020 [1]. The confirmed number of COVID-19 cases in South Africa was 4 million, with an estimated 104,000 deaths at the end of July 2022 [2]. Most healthcare facilities implemented stringent visitation limits in response to the COVID-19 pandemic to stop its spread and safeguard patients, relatives, and healthcare professionals [3,4]. During the COVID-19 pandemic era, the rule requiring limited family member visitation was implemented. By doing this, the severe acute respiratory syndrome coronavirus 2 (SARS-CoV-2) spread was curbed [5,6]. It was confirmed by the WHO [7] that several containment measures, including limiting visits from families to hospitalised patients, should be implemented to preserve safety in healthcare delivery by preventing the transmission of infection, particularly at the hospital level. According to Ning and Slatyer [8], various unique contingency actions were implemented by healthcare organizations to reduce the chain of infection. Hoffman et al. [9] indicated that several public institutions-controlled infections by restricting visitors from hospitals, separating them from hospitalised friends and family to reduce the infection risk. However, Jefferson et al. [10,11] found that the effectiveness or actual impact of this restriction on hospital visits in halting the spread of COVID-19 was not fully understood because there are numerous other potential channels of in-hospital transmission in addition to visits. The known data show that families do not predominately contribute to spreading COVID-19 and other illnesses in hospitals. Therefore, the influence of the family in these transmissions is limited [12,13].

Before the outbreak of COVID-19, visiting hospitalised patients, family members were encouraged to attend for social interaction and emotional support and to enhance family-centred care; however, during the pandemic, visitation was restricted [14]. Fernández-Martnez et al. [15] advocated for family-centred care, respecting the dignity and respect of patients and their families, their cultural beliefs and values, and by delivering accurate and comprehensive information. Evidence suggests that having relatives around when a patient is hospitalised benefits the patient and the care process [16,17,18].

Sagoo and Grytnes [19] reported that the relatives of acutely admitted patients play a vital role in the patient’s treatment and care. Family members are essential in the intensive care unit because critically ill patients rely on them to make surrogate decisions and communicate their needs [20]. Østergaard and Konradsen [21,22] also concur that healthcare practitioners and nurses must pay attention to involving close relatives in planning and completing patient care.

During the COVID-19 pandemic, the policy, restricted entry to the facility, was applied, and only employees accessed the facility. Patients and family members found hospital stay extremely difficult since they were distressed by the unknown circumstances of their loved ones. The COVID-19-pandemic-related visitation restrictions had unforeseen repercussions, such as isolation [23]. Other institutions used stringent rules that limited the number of visitors per period and only let the family come during certain hours of the day [23]. Hospitals were safeguarding staff members (nurses) who care for patients and monitoring them for signs and symptoms of COVID-19 daily. According to research by Chopra et al. [24], family-centred care was in jeopardy during the COVID-19 pandemic, and emotional support was essential for the morale and well-being of patients and healthcare workers.

Patients with COVID-19 may feel loneliness, resentment, anxiety, sadness, and insomnia due to social isolation, perceived risk, uncertainty, physical discomfort, and fear of spreading the virus to others with post-traumatic stress disorder [25]. The unbalanced or unjustified separation of families from their loved ones was risky and further eroded healthcare institutions’ trustworthiness. Family members’ physical presence at the bedside was the mainstay of family-centred care to foster trust, communication, and participation in caregiving and joint decision making [26].

Vhembe district is rural, and the community and family members had limited ways of accessing and interacting with admitted family members physically and virtually. Ashana and Cox [27] believed one could keep a mobile device to continue family-centred care, contact, and communication. Instead of waiting for busy unit clinicians to make the first move, frequent audio or video communication with the patient’s family could be facilitated in their room. The use of smartphones, tablets, and other mobile devices connecting patients and families with streaming cameras was found to be adequate for connecting unit staff with isolated patients, but the situation was not feasible in the Vhembe district due to the area’s rurality and poor connectivity.

The Department of Health changed its visitation policy during COVID-19, and a circular stating “no visitors allowed” was published. As was practiced during COVID-19, restricting family presence to provide emotional support and interaction with loved ones led to a deterioration in the patient’s condition due to stress or feeling abandoned or dumped in the hospital. Even though this was the method used to stop the spread, family members’ experiences were varied, making it difficult for them to communicate with the hospital to learn the status of the patient’s condition. During the execution of the stringent visitation policy, it was unknown how the family members interacted with the admitted member. The study aimed to determine how family members were affected by the performance of a stringent visitation restriction when a patient was hospitalised with COVID-19 at public hospitals in the Vhembe district.

## 2. Setting

**Figure 1:**
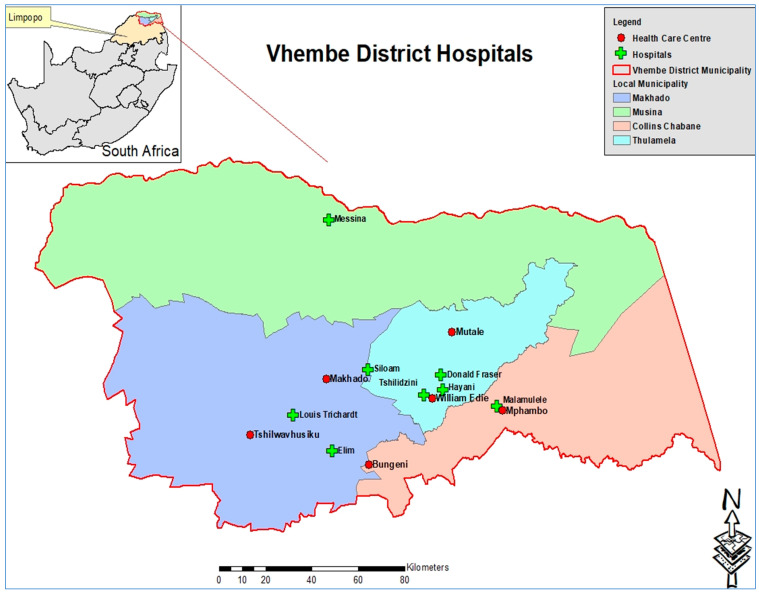
Vhembe district map (Municipal Demarcation Board, 2016 and Community Survey 2016).

The study was conducted in selected hospitals in Vhembe district, which is situated in the northern part of Limpopo Province and shares borders with Capricorn and Mopani districts in the eastern and western directions, respectively. The district covers 21,407 square km of land with a total population of 1,294,722 people, according to Statistics SA (2011). The population is primarily black Africans who are culturally bound and community orientated and believe in collectivism rather than individualism in providing care to a family member, and they value Ubuntu philosophy. Vhembe district consists of four local municipalities, Musina, Collins Chabane, Thulamela, and Makhado, with six district hospitals, Donald Fraser, Elim, Louis Trichardt Memorial, Malamulele, Messina, and Siloam (Figure 1).

## 3. Material and Methods

Qualitative research approach was employed. Following Cresswell and Cresswell [28], an exploratory, descriptive design was followed, a design of inquiring about the lived experiences of individuals about a phenomenon, as described by participants. This description culminates from the essence of the experiences of individuals who have experienced visitation restrictions during the COVID-19 pandemic in district hospitals in Vhembe district.

## 4. Population and Sampling

Brink [29] described a population as the entire group(s) or objects that interest the researcher or meet the criteria the researcher is studying. The sample of this study was family members of patients who were diagnosed with COVID-19 and were admitted to these district hospitals. Purposive sampling of three patients’ records from each hospital was conducted, for those patients who were admitted for more than a week, and the telephone contacts of their relative or family member were recorded. A total of eighteen family members were purposively sampled. However, only twelve family members of patients formed the sample size after the telephonic interview. Data saturation was reached with nine participants, and researchers continued with interviews until the twelfth participant, as there was no new information was coming out. Saturation is used in qualitative research as a criterion for discontinuing data collection and/or analysis [30].

## 5. Data Collection Method

Prior to data collection, approval was sought from relevant institutions; the University of Venda Ethics Committee granted ethical approval (SHS/20/PDC/19/0608). The Limpopo Department of Health Research Committee and the institutions’ Nursing Managers are permitted to enter the health facilities and access the patient address book or register. The participants who gave verbal informed consent and agreed to participate in the study were assured of confidentiality and privacy, that their names or information being recorded would not be linked to their identity. Five female researchers who are holders of PhDs, professional nurses, and work as researchers at the university conducted data collection and analysis. Researchers collected data telephonically to minimize the spread of infection as the country was still under stringent restrictions on movement. Rapport was built with participants to make them feel at ease by letting communication flow in a simple and non-threatening manner, at a time convenient for them. The authors aligned to COREQ guidelines. As in Walsh et al. [31], unstructured in-depth interviews were conducted in the local language and were translated verbatim into English by a language specialist fluent in Tshivenda and Xitsonga. The central question was, “*As a family member of the hospitalised loved one, how was it for you when you were not allowed to visit them?*” The probing questions perceived consequences. Probing was undertaken to gain additional information on specific issues during the interview. Forty-five minutes was allotted for each discussion; however, sometimes, it lasted for an hour when they felt emotionally overwhelmed and were provided with spiritual support. Data were collected in May to July 2021, which was lockdown level three. During this level, movement was restricted unless the person had permission from the security authorities. Participants were interviewed in their local language, which was Tshivenda and Xitsonga. Data were collected until data saturation was reached with twelve family members. A voice recorder was utilized to record all the information provided by the participants, and field notes were also gathered.

### 5.1. Data Analysis

The collected data were transcribed verbatim and translated into English by the linguist. Researchers adapted Creswell and Creswell [28] open coding data analysis. Members of the research team read and reread the translated data for familiarization and derivation of meanings and to produce initial codes. Codes with similar concepts were grouped to form sub-themes and those sub-themes with similar ideas were further clustered to form final themes. All themes were checked against transcripts for appropriateness. Finally, the themes were described and supported with extracts from transcripts.

### 5.2. Measures to Ensure Trustworthiness

The trustworthiness criteria outlined in Guba and Lincoln [32] were applied. Prolonged engagement ensured credibility as interviews lasted for 45–60 min. This was accomplished by building rapport with participants and clarifying descriptions through familiarity, probing, and a voice recorder. Researchers collected data through telephonic in-depth individual interviews and voice recorder to provide the triangulation and confirmability of findings. A member check was carried out to verify and validate the results among the participants.

### 5.3. Presentation of Results

#### Characteristics of Participants

This study was conducted amongst 12 participants who were close family members and visited the admitted relative during lockdown. The characteristics of participants are presented in Table 1.

### 5.4. Presentation of Findings

Three themes with sub-themes (Table 2) emerged, such as inadequate measures for temporary communication channels and techniques, the mental health effects of COVID-19 admission, and poor/lack of standardised visitation policy during the COVID-19 pandemic.

In the key, the participant number was put in brackets (..), and hospitals were presented as follows: Donald Fraser (A), Elim (B), Louis Trichardt Memorial (C), Malamulele (D), Messina (E), and Siloam (F). Below are the highlighted themes and accompanying direct quotes.

**Theme 1:**  **Inadequate measures for temporary communication channels and technique**

The COVID-19 pandemic challenged patient centredness and family-centred care; due to the universal policy to curb the spread of the disease, it negatively impacted patients. COVID-19 created social isolation, infringing on the relative closeness and visual insights important for family members in coping with this stressful situation.

**Sub-theme 1.1:**  **Feeling of helplessness**

During the hospitalization of their loved ones, families frequently feel helpless and experience severe levels of emotional pain.

This was supported by a direct quote from parent participant (4) from hospital D: “*Hey, this policy makes it so difficult for us as a family as we cannot rely on nurse’s report. Especially when you listen to social media, people are dying. Each time you hear your phone ring, you expect bad news*”.

The spouse of participant (10) from hospital E had to say: “*For me to be told that I cannot see my husband while he was so seriously sick was tough for me. Even if it meant that I might die, it would be better to be allowed to see him. Hey, what can we do it was so… terrible*”.

**Sub-theme 1.2:**  **Limited opportunities to communicate with the sick family member**

Patients and family members suffered unforeseen repercussions due to the restrictive visitation regulations instituted in hospitals during the COVID-19 pandemic. Family members complained they had few opportunities to visit and speak with their ill family members.

However, a child participant (5) from hospital E had a positive experience: “*Fortunately, I communicated with him over the phone. I could listen to his*” voice or video call and concluded that he was getting better by the day. The other spouse participant (9) from hospital F who also felt receiving positive communication, said: “*I asked the nurses to call my wife as I didn’t have any phone with me. They called her, and I was able to talk to my wife. On the other hand, my wife says when she sees the call from the hospital… she would be afraid to answer it*”.

The lack of a standardised communication pattern with the patient or healthcare provider was seen as stressful as the relatives initiated the communication. Healthcare institutions should have clear-cut policies or agreements with relatives on admission on *when and how* to communicate.

**Theme 2:**  **Mental health effects of COVID-19 admission**

The COVID-19 pandemic had severe and far-reaching repercussions for mental health, and hospitalization has been identified as a potential risk factor for anxiety or depression. Also, among those who were not hospitalised, it amplified much more severe mental health problems such as post-traumatic stress.

**Sub-theme 2.1:**  **Fear and emotional trauma**

Due to visitation limitations brought on by the COVID-19 pandemic, hospitalized patients’ families experienced anxiety and emotional distress, which impacted family-centred care. Different participants expressed fear of losing their loved ones.

Parent participant (11) from hospital F said: “*I was worried that I could not visit my son to observe how he was improving. I used to wake at night thinking my son is COVID-19 positive, and I was afraid he might die as he could not breathe well*”.

The spouse of participant (6) confirmed this: “*I was so afraid my husband may die. When my phone rang, I was afraid to answer, it was like I will be told that he is no more. I couldn’t eat, clean the house or even bathe, I was alone at home as I was also positive, children were staying with my mom as they tested negative, but with me, there were mild symptoms*”.

Another spouse, participant (7) from hospital A, expressed fear and anxiety by saying, “*My husband was in a bad state as he was unable to use his phone. I was relying on nurses for a report of his condition. His being seriously ill and my inability to see or talk to him killed me. I don’t know how I survived; I only thank the Lord for the counseling I received from my pastor telephonically, and the calls from family members helped so much*”.

The parent participant (3) from hospital F expressed how she suffered emotional trauma when relating a story cited by her admitted child by saying, “*It would be better if the hospital allows one family member to come and visit as one patient died and was asking to see his eldest son, but it was denied. I think he died with a broken heart. I felt bad about that situation*”.

**Sub-theme 2.2:**  **Value of family support**

Limitations could be particularly challenging in the units, where strong family support was valued and where family visits have been significantly liberalized over the last 20 years. Grandparent participant (8) from hospital B pointed out that they received limited support and were not allowed to visit their hospitalised family member. A parent participant expressed little support by saying; “Y*ou know doctor that issue of not allowing visitors to visit the sick family member is a no no no!!!, I don’t like that! As family members, we need support to visit relatives, and our presence means a lot*”.

The same participant further said: *I felt bad that I wasn’t there for my ailing family member’s bedside and that I wasn’t helping with the care. Families must work hard to ensure that their loved ones receive the comfort and dignity they deserve while in the hospital*. The findings support the fact that patients admitted to the hospital during the COVID-19 pandemic experienced fear and anxiety and suffered from the lack of contact with their relatives. Family support is vital for patient treatment, care, and recovery.

**Theme 3:**  **Poor/Lack of standardised visitation policy during COVID-19 pandemic**

The international community has made progress toward preparing for and mitigating the impacts of pandemics. The World Health Organization stipulated specific standards for detecting, reporting, and responding to outbreaks. Despite evidence to the contrary, it appears that hospital visiting restrictions implemented during the COVID-19 pandemic had unexpected repercussions, given the critical role that family visits and involvement play in family-centred treatment.

**Sub-theme 3.1:**  **Mixed feelings regarding visitation**

Given the contagious nature of COVID-19, the Centers for Disease Control and Prevention (CDC) recommended limiting visitors. Different public health institutions established internal policies regarding visitation, so these were not standardised across the country. Some institutions had limited numbers and reduced the visitation time to less than 15 min; some had no visitation but allowed telephone calls to healthcare providers.

Family members provided their different policy experiences and suggested some interventions to accommodate families in the care of hospitalised patients.

Parent participant (3) from hospital D said, “*For the sake of protecting us from getting infected, the policy is good, but this is too much and too painful*”.

The spouse participant (2) from hospital A also displayed a positive understanding when saying, “*Yes, a nurse explained why we were expected not to visit our loved ones. She said visitors were prohibited according to the Government’s COVID-19 policy. But we can call; they will give us the progress*”.

Participants had variable responses regarding the restricted visitation policy practiced in the hospitals. This was confirmed by parent participant (1) from hospital B, saying: “*But on the other hand, the hospital is protecting us from getting infected from other patients or family members. This policy has good and bad results”.*

**Sub-theme 3.2:**  **Suggested visitation interventions**

The child participant (5) from hospital F said, “*I think the hospital was supposed to have a way for us to see our patients. Maybe through the window, or they allow one family member to enter the unit using protective clothes, for those who can afford*”.

Participants elaborated further on the interventions the hospitals could use to involve and communicate with loved ones when hospitalised, isolated, and unable to be physically visited. The child participant (5) from hospital F said*:* “*I think if maybe the hospital can allow us to use their phone and contact our families*”.

However, the spouse participant (6) had a different view, saying, “*If maybe the hospital staff could call the family daily and tell them about the patient’s condition*”.

The spouse participant (12) from hospital B suggested that: “*I called, but she couldn’t talk on the phone, I even assumed that maybe she has passed on and started to cry. The government should allow relatives to see their loved ones through the window and maybe set a specific time for them to do so or make the rotation for the family to go in at a time*”.

Family members play an important role by offering emotional support and participating in shared decision making when one family member is hospitalised. The family members were disappointed as they could not see or talk with their loved ones virtually or through the windows. However, other families appreciated the government in trying to curb the pandemic.

## 6. Discussion

This paper examines the perspective of family members regarding implementing a restricted visitation policy when a family member was admitted with COVID-19 at public hospitals in rural areas. While the government-imposed visitor restrictions were unavoidable during the early stage of the pandemic, the policy was implemented as an infection prevention and reduction strategy as more concerns were around virus transmission to and from visitors [33]. Family members experienced limited communication on the progress of the hospitalised family member. Patients who were critically ill may frequently be incapable, and information was commonly provided to family members. Reduced family presence induced feelings of helplessness and anxiety in the family [15]. Visitation limitations strain healthcare providers because they necessitate more family communication [33]. The humanization process requires effective communication [33,34]. Vhembe district in Limpopo province is rural; villages experience limited technological resources, like poor internet connectivity, to conduct virtual communication. Again, it was noted that most participants did not have smartphones for video calling or video conferencing. The units were overcrowded, and healthcare professionals had a higher workload, experienced moral distress, and had minimal opportunities to call family members or to answer the unit phone to update them on their hospitalised relative’s condition. This notion was confirmed by Au et al. [35], that when nurses are faced with providing bulk informal updates to families, they will communicate less frequently. Sasangohar et al. [36] pointed out that some challenges that hinder constant updates to families were workload and privacy concerns.

There was a reported rise in the need for information and frequent updates on patients’ conditions from healthcare providers when the family could not see their hospitalized relatives [36,37,38]. Health practitioners were, therefore, required to communicate with family members more frequently and in greater detail than when such information could be obtained through direct, in-person interaction [39].

When visitation was restricted, family-centred care was threatened. The essential component of family-centred care is communication. During the COVID-19 era, the method of providing patients with health information and interacting with their families was impacted. Given the significance of family involvement and visiting in patient- and family-centred care, early research, according to Burns et al. [40], suggests that the COVID-19 pandemic’s restrictive visitation restrictions implemented in hospitals will have unforeseen effects on patients, family members, and medical professionals.

Not seeing their hospitalized family member in person caused a demand for additional information and updates on the family member’s condition, as well as anxiety, worry, and despair [37]. Family members suffered anxiety and emotional trauma when the restricted visitation policy was implemented at public hospitals. Creutzfeldt et al. [37] further stated that being prohibited from being present at the hospital led to moral doubts and a sense of failing to care for and defend their loved ones. Many family members reported stress due to uncertainty. Health professionals must be aware of the growing need for expert psychosocial support for clients and loved ones when visiting limitations are put in place.

It has been discovered that having relatives and close friends visit a hospitalized patient or nursing care resident has several beneficial consequences on their health and wellness [41]. Family members who “saw” their loved ones using telecommunications services had favourable experiences [42]. Despite technological advances, it was frequently the case that patients were not well enough to engage in video conferences or chats, which diminished the likelihood of preserving social ties within the family [33]. For social engagement within the family, family members of relatives in nursing homes preferred real, safe meetings over digital ones, such as outside trips or meetings behind glass [43]. However, although it hurt their health, research showed that family members accepted and adhered to the visiting limitations to stop the spread of COVID-19 [37,39].

Numerous medical facilities have attempted to replace in-person visits with digital and technical methods. But there were some restrictions on these kinds of gatherings. In contrast to in-person discussions in palliative care settings, video or telephone encounters with family members resulted in fewer goal revisions [42]. The visiting limits also made it more complicated for family members to adequately comprehend their loved ones’ conditions, making it harder for them to know how things were going and how the care was being given. This is true even with alternatives like video visits [43,44]. Instead of regressing from the family-centred care model, recently established healthcare community standards of care could be achieved by novel family involvement tactics in hospital treatment during the COVID-19 pandemic [19].

### Limitations of the Study

Data were collected using telephone; the interviews were conducted when the COVID-19 pandemic was still under lockdown level 3, with restricted movement and fieldnotes, and observation could not be made as not all participants had smartphones, and limited connectivity also disturbed the interviews. The use of one research method (qualitative) also limited the analysis because triangulation could yield rich findings.

## 7. Conclusions

The current study highlights the substantial stress, anxiety, and uncertainty of family members of COVID-19 patients when they cannot communicate or physically visit their relatives due to lockdown restrictions. While the rationale for the restrictive visitation policy was understood to prevent and limit the spread of COVID-19 amongst those vulnerable and family members, constant communication and provision of support remained essential. Communication between family members or relatives and the healthcare team is essential in alleviating anxiety and reducing stress. Government should be prepared for future health pandemic crises by ensuring that policies are in place for visitation and communication.

### Implications for Practice

Findings from this study may guide decisions regarding visitation rules and communication with hospitalised patients’ relatives during the COVID-19 pandemic and any other future pandemics that may arise, designing digital communication interventions to enable contact between relatives and hospitalised persons or healthcare professionals. A call centre can be opened in the ward/unit as a measure of providing support to relatives and family members.

## Figures and Tables

**Table 1:** Characteristics of participants.

Participant Number	Age	Gender	Ethnic Group	Employed/Unemployed	Relationship
1	63	Male	Venda	Unemployed	Parent
2	30	Male	Venda	Unemployed	Spouse
3	42	Female	Tsonga	Unemployed	Parent
4	28	Female	Venda	Employed	Parent
5	21	Male	Tsonga	Unemployed	Child
6	40	Female	Tsonga	Employed	Spouse
7	28	Female	Venda	Employed	Spouse
8	60	Male	Venda	Unemployed	Grand parent
9	44	Male	Venda	Employed	Spouse
10	65	Female	Venda	Pensioner	Spouse
11	64	Female	Tsonga	Pensioner	Parent
12	55	Male	Venda	Pensioner	Spouse

**Table 2:** Themes and sub-themes as perceived consequences.

Themes	Sub-Themes
1. Inadequate measures for temporary communication channels and techniques.	1.1. Feeling of helplessness 1.2. Limited opportunities to communicate with the sick family member
2. Mental health effects of COVID-19 admission	2.1. Fear and emotional trauma 2.2. Value of family support
3. Poor/ lack of standardised visitation policy during COVID 19-pandemic	3.1. Mixed feelings regarding visitation 3.2. Suggested visitation interventions

## Data Availability

All data supporting this manuscript have been made available. All data transcripts coded and analysed during this study are included in this article.

## Guidelines and Standards Statement

This manuscript was drafted against the Standard for Reporting Qualitative Research (SRQR) [45] and the COREQ guidelines [46].
